# The depletion of PinX1 involved in the tumorigenesis of non-small cell lung cancer promotes cell proliferation via p15/cyclin D1 pathway

**DOI:** 10.1186/s12943-017-0637-4

**Published:** 2017-04-04

**Authors:** Xiao-Peng Tian, Xiao-Han Jin, Mei Li, Wei-Juan Huang, Dan Xie, Jia-Xing Zhang

**Affiliations:** 1 Zhongshan School of Medicine, Sun Yat-Sen University, Guangzhou, China; 2 State Key Laboratory of Oncology in South China, Cancer Center, Sun Yat-Sen University, Guangzhou, China; 3 Department of Pathology, Cancer Center, Sun Yat-Sen University, Guangzhou, China; 4 Department of Oncology, The first Affiliated Hospital, Sun Yat-Sen University, No.58, Zhongshan Second Road, 510080 Guangzhou, China

**Keywords:** PinX1, Non-small cell lung cancer, BMP5, Cell cycle, P15

## Abstract

**Background:**

The telomerase/telomere interacting protein PinX1 has been suggested as a tumor suppressor. However, the clinical and biological significance of PinX1 in human non-small cell lung cancer (NSCLC) is unclear.

**Methods:**

PinX1 gene/expression pattern and its association with NSCLC patient survival were analyzed in cBioportal Web resource and two cohorts of NSCLC samples. A series of in vivo and in vitro assays were performed to elucidate the function of PinX1 on NSCLC cells proliferation and underlying mechanisms.

**Results:**

More frequency of gene *PinX1* homozygous deletion and heterozygote deficiency was first retrieved from cBioportal Web resource. Low expression of PinX1 correlated with smoking condition, histological type, T stage, N stage, M stage and TNM stage, and was an independent predictor for overall survival in a learning cohort (*n* = 93) and a validation cohort (*n* = 51) of NSCLC patients. Furthermore, knockdown of PinX1 dramatically accelerated NSCLC cell proliferation and G1/S transition, whereas ectopic overexpression of PinX1 substantially inhibited cell viability and cell cycle transition in vitro and in vivo. p15/cyclin D1 pathway and BMP5 might contribute to PinX1-associated cell proliferation and cell cycle transition.

**Conclusion:**

The cost-effective expression of PinX1 could constitute a novel molecular predictor/marker for NSCLC management.

**Electronic supplementary material:**

The online version of this article (doi:10.1186/s12943-017-0637-4) contains supplementary material, which is available to authorized users.

## Background

Globally, lung cancer is a major clinical problem, which is one of the leading causes of cancer mortality, resulting in more than 2 million deaths each year [1]. Non-small-cell lung cancer (NSCLC) accounts for about 85% of all lung cancer, with 5 year-survival of less than 20% [2]. Early diagnosis represents one of the most effective strategies in improving survival and prognosis of lung cancer patients [3–5]. Therefore, it is important to understand the molecular mechanism in NSCLC tumorigenesis in order to identify biomarkers for early diagnosis and management of NSCLC.

PinX1 was first identified in a yeast two-hybrid screen as TRF1-binding protein and this genetically conserved nuclear protein was shown to be an endogenous telomerase inhibitor [6–8]. Moreover, it has been demonstrated that PinX1 is a major haplo-insufficient tumor suppressor gene, stemming from its correlation with chromosome instability and cancer initiation [9]. Our group recently reported that not only does PinX1 contribute to telomerase activity and cancer tumorigenicity but it also increases the sensitivity of cancer cells to DNA damage and chemo-radiotherapy [10–12]. In addition, recent studies have shown that PinX1 could suppress cell cycle progression [13–16]. PinX1 was also shown to arrest cell cycle on spindle assembly checkpoint and this associate with its localization during different stage of cell cycle. It in turn influences cancer cell proliferation and taxol sensitivity [17, 18].

Although there is mounting evidence suggesting PinX1’s role in cancer development and progression [19–22]. We have not seen any reports of its involvement in pathogenesis and tumorigenesis of NSCLCs. Here we provided the connection and identified the potential molecular mechanisms for PinX1 to promote NSCLCs. In this study, we first described the expression pattern of PinX1 in human NSCLC tissues. We also demonstrated that low expression of PinX1 correlated with smoking condition, histological type, T stage, N stage, M stage and TNM stage, and was an independent predictor for overall survival in NSCLC patients. Furthermore, the function and mechanisms studies of PinX1 suggest that PinX1-arrested cell cycle transition accounts for the NSCLC’s cell proliferation. In addition, p15/cyclin D1 pathway and BMP5 might contribute to PinX1-associated cell proliferation and cell cycle transition.

## Methods

### Analysis of NSCLC data in *cBioportal for Cancer Genomics* database

The cBioPortal for Cancer Genomics is an open-access downloaded bio-database, providing visualization and analyzing tool for large-scale cancer genomics data sets (http://cbioportal.org). This portal collected records that were derived from 147 individual cancer studies, in which 31 types of cancer were analyzed, which included over 21000 samples [23, 24]. Analysis of the 1788 NSCLC samples (1098 lung adenocarcinoma cases and 682 lung squamous cell carcinoma cases) from this database was performed *in silico*.

### Patients and tissue specimens

Ninety-three NSCLC patients from the Department of Radiotherapy, Cancer Center, Sun Yat-Sen University between January 2008 and December 2014 were assigned as learning cohort. Another 51 NSCLC cases in the same period from the first Affiliated Hospital, Sun Yat-Sen University were assigned as validation cohort. These cases were selected based on the availability of resection tissues and follow-up data. All selected cases had not received preoperative radiation or chemotherapy before diagnosis biopsies. All samples were classified according to the TNM (UICC, 2014) staging and evaluated for histological type by the same pathologist. The study was approved by the Medical Ethics Committee of the two institutions and involved only informed consent patients. The clinical-pathological characteristics of the two cohorts are summarized in Table 1.

**Table 1:** Association of PinX1 expression with patient’s clinicopathological features in NSCLC

Variable	Learning cohort				Validation cohort			
	Cases	Negative expression (%)	Positive expression (%)	*P*-value*	Cases	Negative expression (%)	Positive expression (%)	*P*-value*
Age(years)								
<56^a^	42	21(50%)	21(50%)	0.068	22	13(59.1%)	9(40.9%)	0.443
≥56^a^	51	35(68.6%)	16(31.4%)		29	14(48.3%)	15(51.7%)	
Gender								
Male	61	39(63.9%)	22(36.1%)	0.312	35	17(48.6%)	18(51.4%)	0.355
Female	32	17(53.1)	15(46.9%)		16	10(62.5)	6(37.5%)	
Smoking Condition								
Smoker	42	32(76.2%)	10(23.8%)	0.004	20	16(80%)	4(20%)	0.002
Nonsmoker	51	24(47.1%)	27(52.9%)		31	11(35.5%)	20(64.5%)	
WHO grade								
G1	28	18(64.3%)	10(35.7%)	0.708	16	9(56.2%)	7(43.8%)	0.843
G2	37	22(62.2%)	15(37.8%)		20	11(55%)	9(45%)	
G3	28	16(57.1%)	12(42.9%)		15	7(46.7%)	8(53.3%)	
Histological Type								
Squamous cell carcinoma	37	29(78.4%)	8(21.6%)	0.002	22	16(72.7%)	6(27.3%)	0.046
Adenocarcinoma	46	25(54.3%)	21(45.7%)		23	9(39.1%)	14(60.9%)	
Others	10	2(25%)	8(75%)		6	2(33.3%)	4(77.7%)	
T stage								
T1-T2	38	15(39.5%)	23(60.5%)	0.001	18	4(22.2%)	14(88.8%)	0.001
T3-T4	55	41(74.5%)	14(25.5%)		33	23(69.7%)	10(30.3%)	
N stage								
N0	26	9(34.6%)	15(57.7%)	0.005	16	5(31.2%)	11(68.8%)	0.036
N1-N3	67	47(70.1%)	22(29.9%)		35	22(62.9%)	13(37.1%)	
M stage								
M0	66	45(68.2%)	21(31.8%)	0.014	35	14(40%)	21(60%)	0.006
M1	27	11(40.7%)	16(59.3%)		16	13(81.2%)	3(18.8%)	
TNM stage								
I-II	25	6(24%)	19(76%)	0.000	14	4(28.6%)	10(71.4%)	0.032
III-IV	68	50(73.5%)	18(26.5%)		37	23(62.2%)	14(37.8%)	

*Chi-square test; ^a^Mean age; *T* tumor, *N* node, *M* metastases

### Immunohistochemistry (IHC)

Slides were dried overnight at 37 °C, dewaxed in xylene, rehydrated with graded alcohol, and immersed in 3% hydrogen peroxide for 20 min to block endogenous peroxidase activity. For antigen retrieval, tissue slides were boiled in tris (hydroxymethyl) aminomethane-EDTA buffer (pH 8.0) in a pressure cooker for 10 min. The slides were incubated with 10% normal rabbit serum at room temperature for 20 min to reduce nonspecific interactions. Subsequently, tissue slides were incubated with anti-PinX1 antibody (1:200, ProteinTech Group, Inc.) for 60 min at 37 °C in a moist chamber. After five rinses with 0.01 mol/L phosphate-buffered saline (PBS, pH = 7.4) for 10 min, the slides were incubated with a secondary antibody (Envision, Dako, Glostrup, Denmark) at a concentration of 1:100 for 30 min at 37 °C, followed by PBS washes and finally stained with DAB (3,3-diaminobenzidine). The nucleus was counterstained with Meyer’s hematoxylin. PBS alone was used as a negative control.

### Immunohistochemistry evaluation

PinX1 immunoreactivity was classified by receiver-operator curve (ROC) analysis: (1) low expression defined as less than 65% PinX1 positive cells and (2) high expression defined as greater than 65% PinX1 positive cells. BMP5 positive staining was also divided into low expression cases (cases with score 0–6) and high expression (cases with scores 8–12). (See Additional file 1: Supplementary Materials and Methods).

### Quantitative real-time polymerase chain reaction (qRT-PCR) analysis

The construction of PinX1 and GAPDH sense/antisense primers has been previously described [10]. RNA was reverse-transcribed using SuperScript First Strand cDNA System (Invitrogen, USA) according to the manufacturer’s instructions. qRT-PCR was performed using Real-time PCR system (Applied Biosystems, USA) as follows: 50 °C for 2 min, 95 °C for 10 min, 40 cycles of 95 °C for 15 s, and 60 °C for 60 s. The relative levels of gene expression were represented as ΔCt = Ct_gene_- Ct_reference_, and the fold change of gene expression was calculated by the 2^-ΔΔCt^ Method.

### Cell lines and recombinant lentiviral vector construction

H125, A549, SK-MES-1 and H1299 cells were maintained in DMEM and/or RPMI 1640 supplemented with 10% fetal bovine serum and 1% penicillin–streptomycin at 37 °C in 5% CO_2_. The PinX1 cDNA was cloned into the pCDH-CMV-EF1-copGFP lentivector (System Biosciences, Mountain View, CA, USA). The PinX1-shRNA lentivirus vector has been previously described [10, 11, 16, 20]. The PinX1siRNA transient transfection (GGAGCTACCATCAATAATG) was designed to decrease PinX1 expression temporary.

### MTT proliferation assay

Cellular viability was measured using the MTT proliferation assay (Sigma) according to the manufacture’s protocol. In brief, 1000 cells were seeded in 96-well plates and cultured/treated for 24 h. Viability was measured at different time points from 12 h to 72 h after post-treatment on the basis of experimental requirement.

### Colony forming assay and Western blot analysis

Approximately 500 cells were seeded in each well of a six plate for 24 h. The media was then replaced with fresh RPMI1640 or DMEM containing 10% FBS and the cells were maintained for two weeks. Colonies were fixed with methanol and stained with 0.1% crystal violet in 20% methanol for 15 min. Western blot methods were performed with standard procedure [18]. Details may be found in the Additional file 1: Supplementary materials and methods.

### EdU incorporation

EdU is a thymidine analog whose incorporation can be used like BrdU to label cells undergoing DNA replication. Cells were performed using Cell-Light™ EdU Apollo®488 In Vitro Imaging Kit (Ribobio, Guangzhou China) according to the manufacture’s protocol. The EdU positive cells (red cells) were counted using Image-Pro Plus (IPP) 6.0 software (Media Cybernetics, Bethesda, MD, USA). The EdU incorporation rate was expressed as the ratio of EdU positive cells to total DAPI positive cells (blue cells).

### Flow-cytometry analysis and Cell cycle antibody array

The cells were collected, washed with PBS three times and fixed overnight with cold 70% clod ethanol at 4 °C. The fixed cells were analyzed with flow cytometry (Becton Dickinson, San Jose, CA, USA) using the Alexa Fluor 488 Annexin V/Propidium iodide Apoptosis Assay kit (Invitrogen) following the manufacturer’s instructions.

Total lysates obtained from A549 cells and with or without PinX1 siRNA were used to perform a Cell Cycle Antibody Array according to the manufacture’s protocol (Full Moon BioSystems, Inc., Sunnyvale, CA, USA).

### Xenograft assay

According to institutional guidelines, female 5-week-old athymic nude mice were used for in vivo experiments. Cells (5 × 10^6^ A549-vector, 5 × 10^6^ A549-PinX1 shRNA, 5 × 10^6^ SK-MES-1, 5 × 10^6^ SK-MES-1 PinX1) were injected into the lateral aspect of the rear leg. The tumor diameters were measured with calipers every 4 days, and tumor volumes were calculated (width^2^ × length/2).

### Statistical analysis

All statistical analysis was performed using SPSS software (SPSS Standard v 17.0). The associations between PinX1 expression and clinical-pathological features were assessed with the Chi-square test. Receiver–operator curve (ROC) analysis was performed to determine the cut-off scores for the PinX1 positivity. Survival curves were plotted by Kaplan-Meier analysis and compared by the log-rank test. Multiple Cox proportional hazards regression analysis was carried out to assess the significance of variables for survival. The correlation between expression of PinX1 and BMP5 was performed with the Fisher’s exact test. Data derived from cell line and xenografts experiments are presented as mean ± SE and assessed by the student’s test. *P* values of <0.05 were considered significant.

## Results

### Deletion of *PinX1* gene and decreased expression of PinX1 in NSCLC patients

**Fig. 1:**
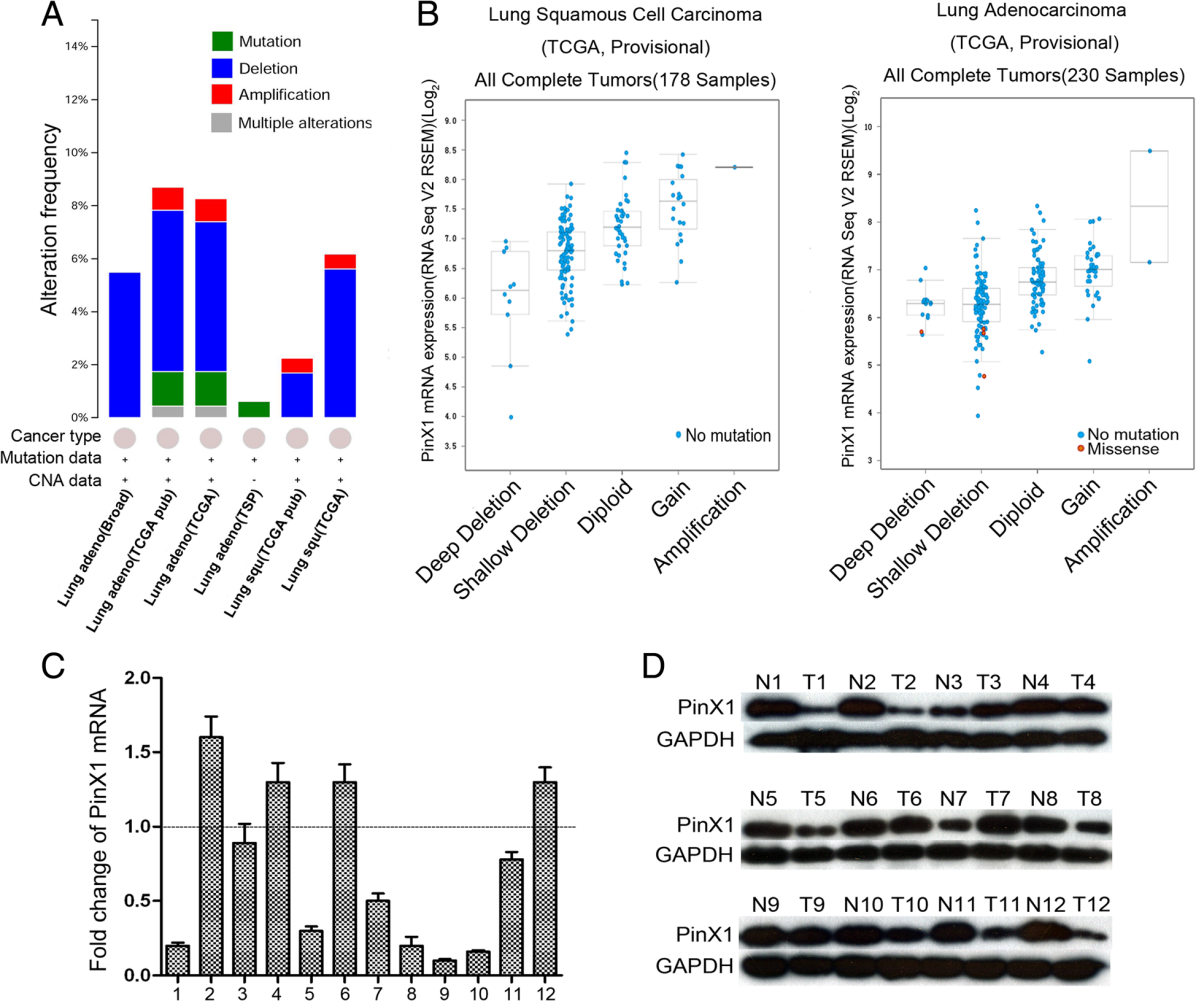
The alterations of *PinX1* gene in cBioportal Web resource online and PinX1 expression in 12 primary NSCLC tissues. **a** The alterations of *PinX1* gene were explored in six NSCLC database (Lung Adenocarcinoma – Broad, Cell 2012; TCGA, Provisional; TCGA, Nature, 2014; TSP, Nature 2008; Lung Squamous Carcinoma – TCGA, Provisional; TCGA, Nature 2012). **b** Relative expression level as a function of relative copy number of *PinX1* genes were plotted in two NSCLC database respectively (Lung Squamous Carcinoma – TCGA, Provisional; Lung Adenocarcinoma –TCGA, Provisional). Deep Deletion — homozygously deleted; Shallow Deletion — heterozygously deleted; Diploid — two alleles present; Gain — low-level gene amplification event; Amplification — high-level gene amplification event. **c** Decreased expression of PinX1 mRNA was examined by qRT-PCR in 8/12 NSCLC cases, when compared with adjacent normal lung tissues. Expression levels were normalized for GAPDH. These samples (12 NSCLC samples) were randomly picked from the learning cohort. Error bars, SD, calculated from three parallel experiments. **d** Decreased expression of PinX1 protein was detected by western blotting in 7/12 NSCLC cases, when compared with adjacent normal lung tissues. These samples (12 NSCLC samples) were randomly picked from the learning cohort. N, adjacent normal lung tissues; T, primary NSCLC samples

The bioinformatics analysis was first performed to detect *PinX1* gene alterations in NSCLC, using cBioportal Web resource online (cBioportal for Cancer Genomic). The deletion of *PinX1* gene accounted for the most alterations and was visualized in six NSCLC database (Lung Adenocarcinoma – Broad, Cell 2012; TCGA, Provisional; TCGA, Nature 2014; TSP, Nature 2008; Lung Squamous Carcinoma – TCGA, Provisional; TCGA, Nature 2012) (Fig. 1a). There were more *PinX1* homozygous deletions and *PinX1* heterozygous deficiencies and less *PinX1* amplifications in two specific NSCLC datasets (namely Lung Squamous Carcinoma – TCGA, Provisional and Lung Adenocarcinoma –TCGA, Provisional) (Fig. 1b).

To verify the results observed from historical data on cBioportal for Cancer Genomic, we selected primary NSCLC tissue samples from our affiliated hospitals. In these selected primary NSCLC tissues, PinX1 mRNA expression was observed in 8 out of 12 (66.7%) NSCLC samples as compared to their normal counterparts by qRT-PCR (Fig. 1c). Western blot analyses revealed that 7 out of 12 (58.3%) NSCLC samples had decreased PinX1 expression, when compared with adjacent normal lung tissues (Fig. 1d).

### PinX1 associated with NSCLC patients’ clinicopathological features

**Fig. 2:**
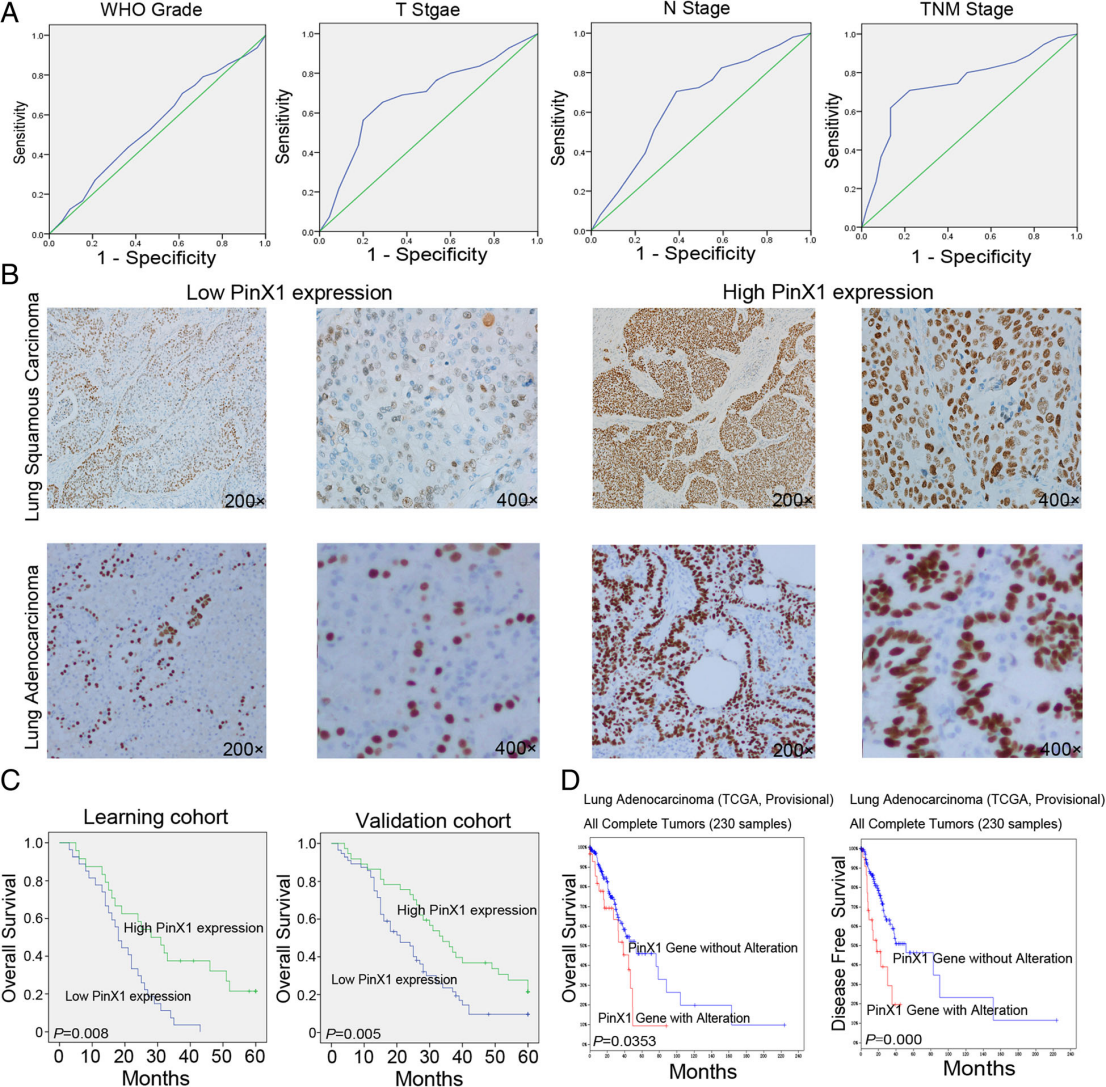
PinX1 expression in tissues obtained from two NSCLC cohorts and its prognostic significance in NSCLC patients. **a** Receiver operation characteristic curve analysis was employed to determine the cut-off score for the overexpression of PinX1. The sensitivity and specificity for each outcome were plotted. **b** Representative immunohistochemical images show the overexpression of PinX1 and low expression of PinX1 in a NSCLC tissue. high PinX1 expression in Lung Squamous Carcinoma (200×, 400×, upper right); high PinX1 expression in Lung Adenocarcinoma tissue (200×, 400×, lower right); low PinX1 expression in Lung Squamous Carcinoma tissue (200×, 400×, upper left); low PinX1 expression in Lung Adenocarcinoma tissue(200×, 400×, lower left). **c** High expression of PinX1 was associated with poor prognosis of NSCLC patients. Kaplan–Meier plots show the Overall Survival curves of 93 ESCC patients in the learning cohort and 51 NSCLC patients in the validation cohort, according to PinX1 expression levels in the primary tumor (*p* < 0.05, log-rank test). **d** *PinX1* gene with alteration was correlated with Overall Survival and Disease-Free Survival in cBioportal Web resource online (Lung Adenocarcinoma – TCGA, Provisional)

Immunohistochemistry was utilized to examine the expression of PinX1 in NSCLC patients (Fig. 2b). According to the ROC curve (Fig. 2a, Additional file 2: Table S1 and Additional file 3: Figure S1), low expression of PinX1 was discovered in 56/93 (60.2%) in the learning cohort and 27/51 (52.9%) in the validation cohort. As shown in Table 1, the association between PinX1 expression and the clinical-pathological parameters in learning cohort and validation cohort suggested that low expression of PinX1 is significantly associated with NSCLC patients’ smoking condition, histological type, T stage, N stage, M stage and TNM stage. However, no significant associated between PinX1 expression and other clinical-pathological features, such as age, gender, WHO grade (Table 1).

### PinX1 serves as an independent indicator for NSCLC patients’ poor survival

In univariate analysis, Kaplan-Meier analysis also demonstrated a significant impact of well-known clinicopathological prognostic parameters, such as Smoking Condition (*P* = 0.000 and 0.042, respectively), T stage (*P* = 0.000 and 0.009, respectively), N stage (*P* = 0.000 and 0.011, respectively), M stage (*p* = 0.000 and 0.004, respectively), TNM stage (*P* = 0.003 and 0.008, respectively) on NSCLC patient’s survival in both cohorts (Table 2). However, these parameters, such as Age (P = 0.199 and 0.899, respectively), Gender (P = 0.996 and 0.385, respectively), Histological Type (P = 0.293 and 0.252, respectively) had no correlation with NSCLC patient’s survival in both cohorts (Table 2). Furthermore, low PinX1 staining was correlated with poor survival of NSCLC patient in the learning cohort (media 21 months versus 34 months, *P* = 0.008) and in the validation cohort (media 18 months versus 34 months, *P* = 0.005) (Fig. 2c). In addition, PinX1 gene alteration also correlated with overall survival (media 38.47 months versus 52.56 months, *P* = 0.0353) and Disease-free survival (media 17.0 months versus 51.51 months, *P* = 0.000), which is explored in cBioportal Web resource online (Fig. 2d). In multivariate analysis, the expression of PinX1 expression was found to be an independent prognostic factor for NSCLC patients’ survival in the learning cohort (*P* = 0.046) and in validation cohort (*P* = 0.023) (Table 3).

**Table 2:** Clinical pathological parameters and expression of PinX1 for prognosis of NSCLC patients by univariate survival analysis (Long-rank test)

Variable	Learning cohort				Validation cohort			
	Cases	Mean survival (months)	Media survival (months)	*P*-value*	Cases	Mean survival (months)	Media survival (months)	*P*-value*
Age(years)								
<56^a^	42	27.6	25	0.199	22	26.3	21	0.899
≥56^a^	51	31.8	29		29	25.6	22	
Gender								
Male	61	29.4	27	0.996	35	24.2	19	0.385
Female	32	29.7	26		16	29.6	22	
Smoking Condition								
Smoker	42	22.5	18	0.000	20	20.1	17	0.042
Nonsmoker	51	35.4	33		31	29.7	27	
WHO grade								
G1	28	28.1	21	0.942	16	17.5	14	0.012
G2	37	30.3	27		20	26.4	22	
G3	28	29.8	32		15	34.2	29	
Histological Type								
Squamous cell carcinoma	37	26.5	21	0.293	22	21.3	18	0.252
Adenocarcinoma	46	29.8	27		23	28.2	24	
Others	10	36.5	32		6	35.2	27	
T stage								
T1-T2	38	39.1	37	0.000	18	31.6	31	0.009
T3-T4	55	22.4	16		33	23.3	19	
N stage								
N0	26	43.4	49	0.000	16	35.6	27	0.011
N1-N3	67	24.1	21		35	21.4	18	
M stage								
M0	66	35.6	34	0.000	35	30	25	0.004
M1	27	15	12		16	17	15	
TNM stage								
I-II	25	38.9	38	0.003	14	36.5	27	0.008
III-IV	68	26	24		37	21.8	18	
PinX1 expression								
Negative	56	24.8	21	0.008	27	19.4	18	0.005
Positive	37	36.2	34		24	32.6	28	

*Long-rank test ^a^Mean age; *T* tumor, *N* node, *M* metastases

**Table 3:** Multivariate Cox regression analysis for NSCLC patients

Variable	Learning cohort			Validation cohort		
	HR	95% Cl	*P*-value	HR	95% Cl	*P*-value
PinX1 expression	0.577	0.335-0.991	0.046	0.441	0.191-0.882	0.023
T stage	2.061	1.222-3.482	0.007	5.434	2.162-13.660	0.000
N stage	2.590	1.340-5.009	0.005	2.392	1.113-5.141	0.000
M stage	4.886	2.766-8.630	0.000	3.485	1.777-6.837	0.000
TNM stage	2.725	1.330-5.584	0.006	2.743	1.331-5.650	0.006

### PinX1 suppresses cell proliferation and clonogenicity of NSCLC cell lines in vitro

**Fig. 3:**
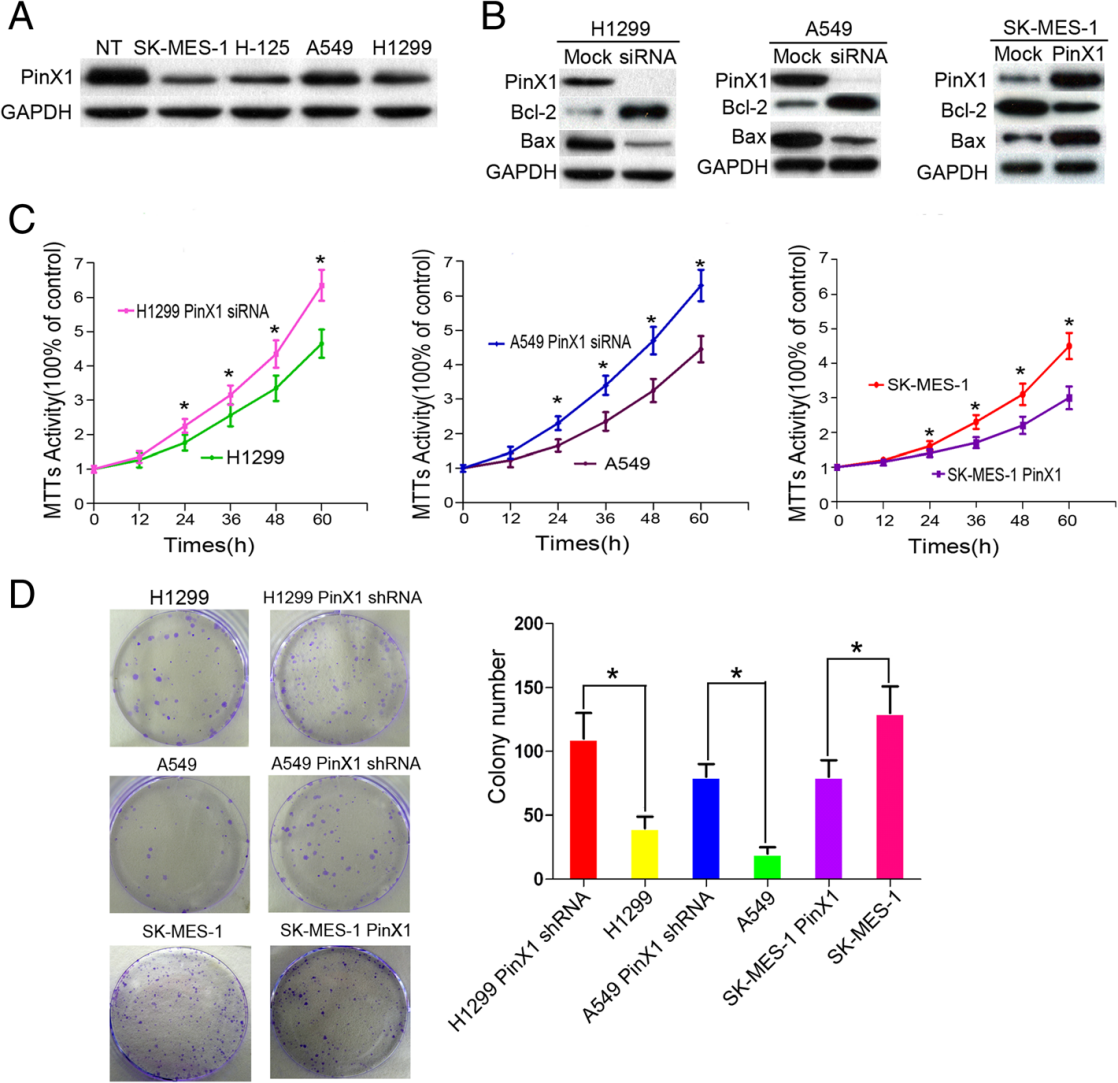
PinX1 suppresses cell proliferation and clonogenicity in NSCLC cell lines. **a** Western blotting analysis showed that the endogenous levels of PinX1 in four NSCLC cell lines (SK-MES-1, H125, A549, H1299) were lower than that in control normal lung tissues. NT, normal lung tissues. **b** PinX1 siRNA was introduced into two NSCLC cell lines, H1299 and A549. pCDH-PinX1 lentiviral were transuded into SK-MES-1. PinX1, Bcl-2, and Bax protein levels were assessed by using western blot analysis. **c** MTT assay was performed to measure viability of cells expressing different levels of PinX1. **d** Colony-forming assay was used to observe colony forming ability of cells expressing different levels of PinX1. Each bar represents the mean ± SD of three independent experiments. *, compared to control group (*P* < 0.05)

We continued our study in vitro by investigating how PinX1 levels correlate with NSCLC cell survival. Of the four NSCLC cell lines (SK-MES-1, H125, A549, H1299) analyzed by Western blot, and all four lines showed lower levels of endogenous PinX1 than those in normal control lung tissues (Fig. 3a). Next, we choose to decrease the PinX1 expression in A549 and H1299, which have higher PinX1 expression among the four cell lines. Meanwhile, we further increased PinX1 expression in SK-MES-1 which has the lowest PinX1 expression. We examined Bcl-2 and Bax protein levels, and discovered that the ratio of Bcl-2/Bax expression was substantially enhanced in PinX1-silenced SK-MES-1 cells and reduced in PinX1-expression A549 and H1299 cells (Fig. 3b). A subsequent MTT analysis and colony-forming assay showed that both A549 and H1299 cells transfected with PinX1 siRNA and/or PinX1 displayed a substantial increase in cell proliferation and/or clone capacity compared with control cells after PinX1 knockdown, while the reintroduction of PinX1 in SK-MES-1 cell line suppressed the cell viability and/or clone ability (Fig. 3c and d). In addition, we also observed that knocked-down PinX1 expression in BEAS-2B cells (Normal lung epithelial cells), the survival capacity of cells was substantially enhanced, and BEAS-2B transfected with PinX1 displayed a substantial drop compared with that of control cells (Additional file 4: Figure S2).

### PinX1 arrested G1/S phase transition of the cell cycle

**Fig. 4:**
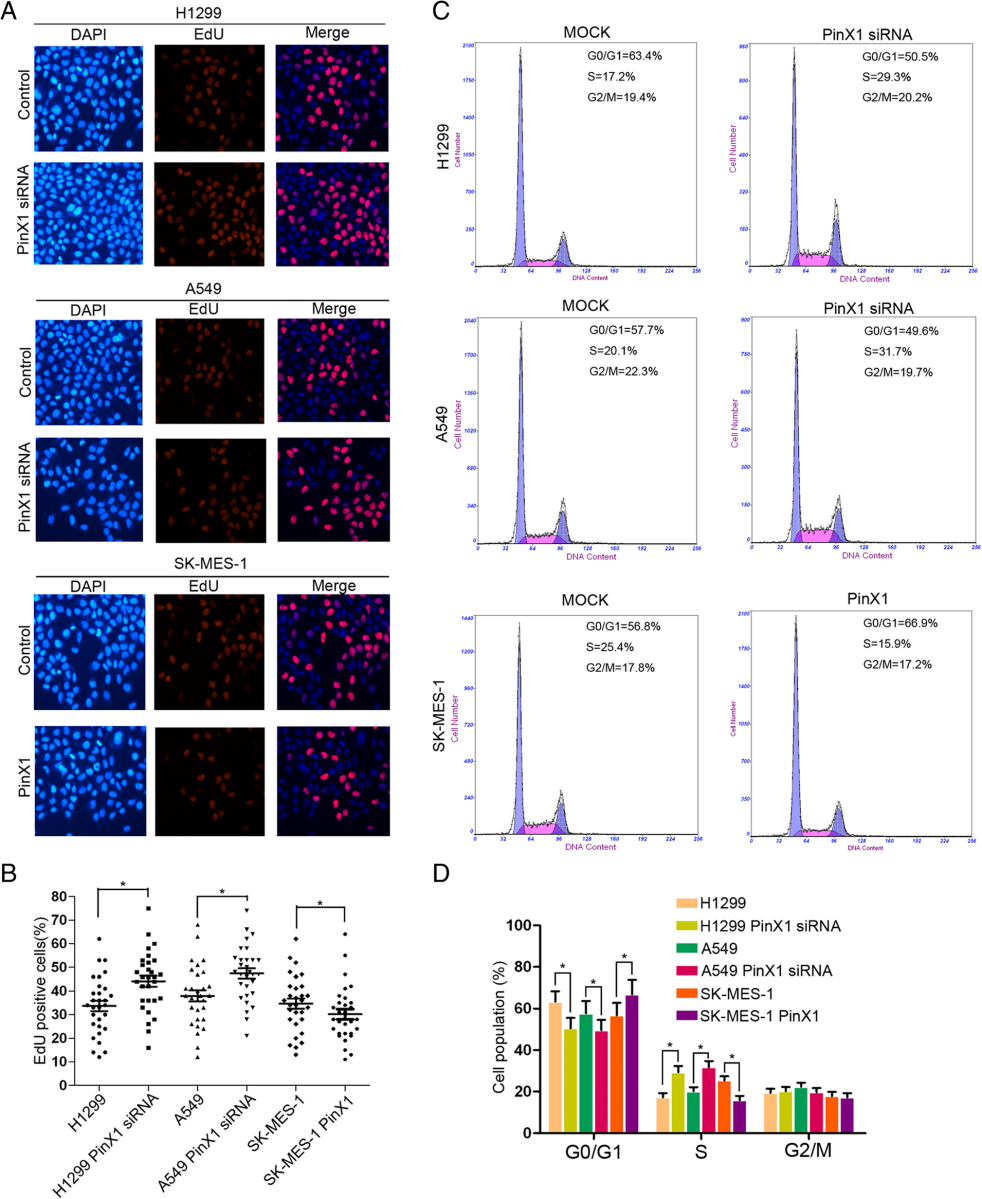
PinX1 arrestd G1/S phase transition of the cell cycle. **a** EdU incorporation assay was used to analyze the population of S phase cells expressing different levels of PinX1. Nuclei were stained with DAPI (blue). S phase cells were stained with EdU (red). Merged images show the percentage of S phase in NSCLC cells. **b** EdU positive cells were represented using scattergram in NSCLC cells expressing different levels of PinX1. At least 300 total cells from triplicate experiments were counted for each group. *, compared to corresponding control group (*P* < 0.05). **c** Cell cycle phases in NSCLC cells expressing different levels of PinX1 were determined by flow cytometry assay. **d** G1/G0, S, and G2/M phase were plotted in NSCLC cells using histogram. *, compared to corresponding control group (*P* < 0.05)

According to our previous study, we asked whether down-regulation of PinX1 influenced the cell cycle in G1/S phase. An EdU cooperation assay and flow cytometry were used to analyze the potential mechanisms of PinX1 suppresses NSCLC cells proliferation. As shown in Fig. 4a and b, the EdU positive cells were dramatically boosted in the both PinX1-silenced cells and significant reduced in PinX1-overexpression cells compare with that control cells. Knockdown of PinX1 resulted in a decrease in the percentage of cells in the G0/G1 phase from 63.4%, 57.7% in control cells to 50.5%, 49.6% in H1299 and A549 cells lines transfected with PinX1 siRNA respectively. Additionally, the population of cells in S phase also displayed the same trend from 17.2%, 20.1% in control cells to 29.3%, 31.7% in PinX1-slienced cells. Simultaneously, an increase in the percentage of cells in G0/G1 phase from 56.8% in control cells to 66.9% and S phase from 25.4% in control cells to 15.9% in PinX1-overexpression cells (Fig. 4c and d). These findings indicated that PinX1 in blocking G1 to S phase transition might contribute to NSCLC cells survival ability.

### PinX1 correlated P15 and cyclinD1 expression in NSCLC cells

**Fig. 5:**
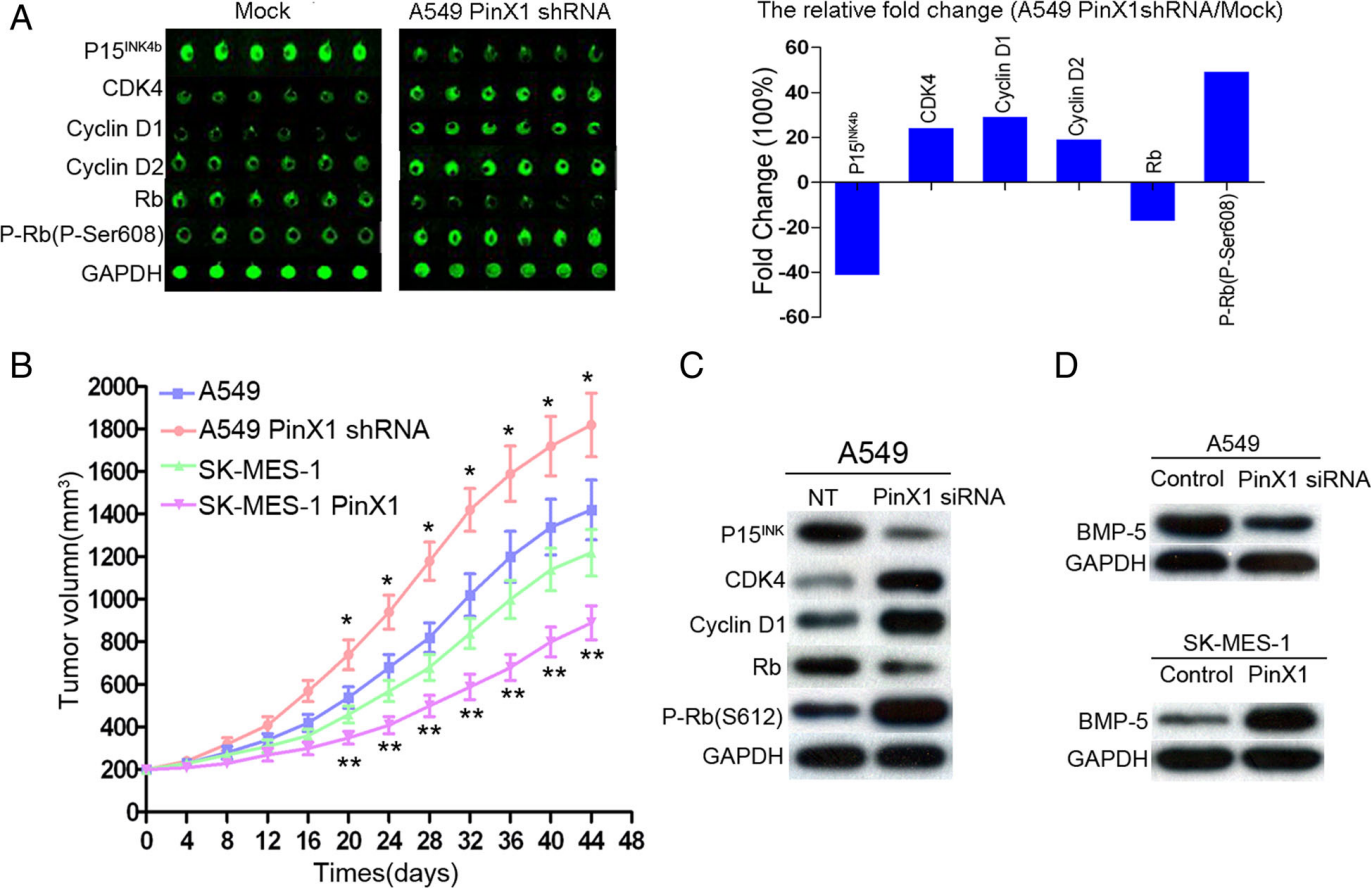
PinX1 inhibit xenograft tumor growth in vivo and target moleculars in NSCLC cells cycle transition. **a** The six proteins, P15^INK4b^, CDK4, CyclinD1, CyclinD2, Rb, and the p-Rb(P-Ser608) were examined in PinX1-siRNA A549 cells compared with that of empty vector by using Cell Cycle Antibody Array (Full moon BioSystems) (left panel). The relative fluorescence intensity fold change (A549 PinX1 shRNA/Mock) was displayed using histogram (right panel). **b** The tumor volume of xenografts was measured calipers every 4 days for 40–46 days. The values represent mean tumor volume ± SE. *, compare with A549 group (*P* < 0.05), ** compare with SK-MES-1 group (*P* < 0.05). **c** Downregulation of P15^INK4b^, Rb and upregulation of CDK4, CyclinD1, p-Rb(p-Ser608) were observed in PinX1-slienced A549 cells by western blotting. **d** The expression of and BMP5 were detected in PinX1-slienced A549 cells and SK-MES-1 PinX1 cells

To identify the molecular basis of PinX1 regulation of target proteins in NSCLC cells transition from G0/G1 phase to S phase, the expression of several relevant activators or inhibitors of the cell cycle were detected by antibody microarrays and western blot assay in PinX1-silenced A549 cells. In this antibody arrays, we discovered the expression of P15^INK4b^ and Rb decreased 0.4-, 0.18- fold and the expression of CDK4, CyclinD1, CyclinD2, p-Rb(p-Ser608) were increased 0.25-, 0.31-, 0.2-, 0.5- fold in PinX1-slienced A549 cells (Fig. 5a). Furthermore, western blot also confirmed that decreased P15^INK4b^ and increased CDK4, CyclinD1, p-Rb(P-Ser608) expression were observed in PinX1-slienced A549 cells (Fig. 5c). These results indicated that P15/cyclinD1 pathway might contribute to the PinX1-arrested G0/G1 to S phase transition.

### PinX1 silencing increases xenograft tumor growth in vivo

Xenograft assay was used to investigate if PinX1 knockdown could stimulate NSCLC cells proliferation in vivo. As shown in Fig. 5b, tumors from nude mice injected with A549-PinX1shRNA grew much faster and weighed significantly more at day 44 (mean tumor volume of 200 mm^3^ to 1820 ± 155 mm^3^), compared to control A459 tumors (mean tumor volume of 200 mm^3^ to 1420 ± 138 mm^3^, *P* < 0.05). Furthermore, the growth of the SK-MES-1-PinX1 tumors was substantially suppressed (a mean tumor volume of 1220 ± 116 mm^3^), compared with that in control SK-MES-1 tumors (a mean tumor volume of 896 ± 95 mm^3^, *P* < 0.05).

### PinX1 associated with cell proliferation and cell cycle transition relies on BMP5 in NSCLC cell lines

**Fig. 6:**
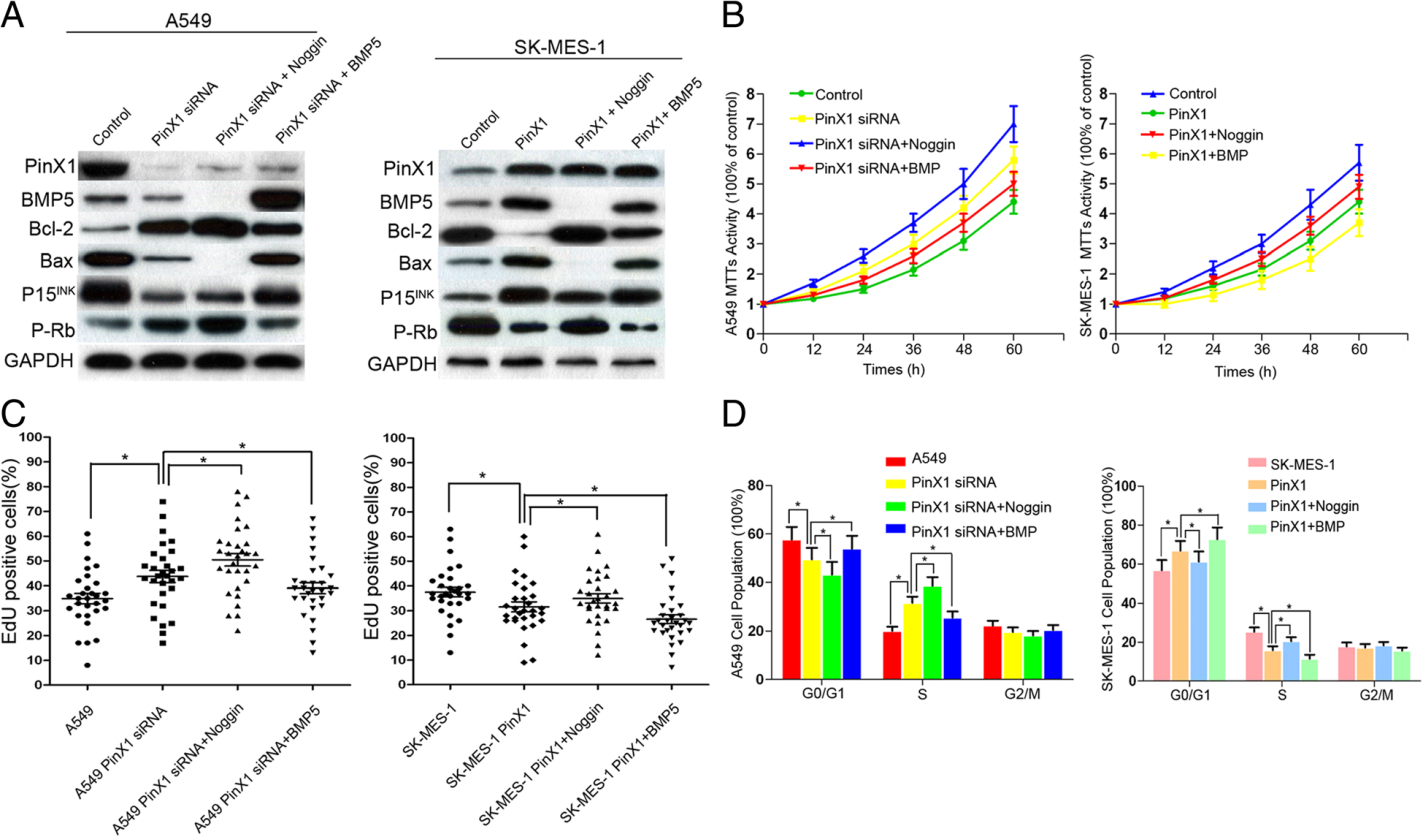
PinX1 associated with cell proliferation and cell cycle transition rely on BMP5 in NSCLC cell lines. **a** Western blotting was used to detect the expression of PinX1, BMP5, Bcl-2, Bax, P15^Ink^and p-Rb in A549 PinX1-silenced and SK-MES-1-PinX1 cells treated with noggin (0.5 mg/ml) and BMP5 (10 μg/ml). **b** Cell viability treated with noggin (0.5 mg/ml) and BMP5 (10 μg/ml) was measured by MTT assay in A549 PinX1-silenced and SK-MES-1-PinX1 cells. **c** The percentage of S phase in A549 PinX1-silenced and SK-MES-1-PinX1 cells treated with noggin (0.5 mg/ml) and BMP5 (10 μg/ml) were determined by EdU cooperation assay. *, *P* < 0.05. **d** G1, G2, and S cell cycle phases in A549 PinX1-silenced and SK-MES-1-PinX1 cells treated with noggin (0.5 mg/ml) and BMP5 (10 μg/ml) were determined by flow cytometry assay. *, *P* < 0.05

According to another study, the differentially expressed mRNAs were identified by mRNA microarray analysis in PinX1 overexpressed group. The fold change (14.54) of BMP5 was significantly increased in microarray analysis and was expressed at the highest levels [25]. Therefore, BMP5 was involved in PinX1-slienced A549 cells and PinX1-overexpressed SK-MES-1 cells to understand the possible molecules of PinX1 act on P15/cyclinD1 pathway. As it shown in Fig. 5d, BMP5 expression was significantly decreased in PinX1-slienced A549 cells, and increased in PinX1-overexpressed SK-MES-1 cells. As it shown in Fig. 6a, Bcl-2/P-Rb expression was up-regulated in PinX1-slienced A549 cells and was down-regulated in PinX1-overexpressed SK-MES-1 cells. Bax/P15^INK^ expression was decreased in PinX1-slienced A549 cells and increased in PinX1-overexpressed SK-MES-1 cells. After introducing BMP5-inhibitor noggin, Bcl-2/P-Rb expression was increased and Bax/P15^INK^ expression was decreased in PinX1-overexpressed SK-MES-1 cells. Moreover, Bcl-2/P-Rb expression was decreased and Bax/P15^INK^ expression was increased in PinX1-slienced A549 cells by BMP (10 μg/ml) treatment (Fig. 6a). Next, MTT analysis, flow cytometry and EdU cooperation assay further demonstrated that the function of PinX1 in cell proliferation was inhibited by noggin and restored by BMP-5 (Fig. 6b, c and d). Together, these results support the notion the BMP5 may be important for PinX1 mediated cell cycle and its expression may be related to NSCLC proliferation.

### Correlation between the expression of PinX1 and BMP5 in NSCLC patients

Utilizing the previously criterions of a semi-quantitative scale evaluation, low expression of BMP5 was observed in 41/93 (44.1%) in the learning cohort and 23/51 (45.1%) in the validation cohort. Further correlation analysis demonstrated that a prominently consistency correlation between PinX1 expression and BMP5 in both cohorts (*P* = 0.015 in learning cohort and *P* = 0.006 in validation cohort, Fisher’s exact test, Table 4).

**Table 4:** Correlation between expression of PinX1 and BMP5 in NSCLC patients

	Learning cohort				Validation cohort			
	Cases	BMP-5 Low expression (%)	BMP-5 High expression (%)	*P*-value*	Cases	BMP-5 Low expression (%)	BMP-5 High expression (%)	*P*-value*
PinX1 Negative expression	56	31(33.3%)	25(26.9%)	*P* = 0.015 (*r* = 0.252)	27	17(33.3%)	10(19.6%)	*P* = 0.006 (*r* = 0.381)
PinX1 Positive expression	37	10(10.8%)	27(29%)		24	6(11.8%)	18(35.3%)	

*Spearman correlation analysis indicated that PinX1 and BMP5 expression levels were consistently correlation (Fisher’s exact test)

## Discussion

PinX1 is a newly cloned gene that has been mapped to chromosome 8P23.1 [26]. However, the molecular status of *PinX1* and its attendant expression patterns are vastly different in various tissues and tumors types. This variation suggests that abnormal gene regulation and/or protein functions of PinX1 in tumorigenesis are complicated and are likely to be tumor-type-specific [27–29].

Our initial observations focused on the incidence of *PinX1* alteration frequency in NSCLC from cBioportal Web resource online [30]. In agreement with previously studies, the PinX1 mRNA Sqe analysis demonstrated that the frequency of gene *PinX1* homozygous deletion occurs in NSCLC tumorigenesis [19, 26, 31]. Western blotting and qRT-PCR assay also demonstrated that the protein and mRNA expression of PinX1 was decreased in 12 primary NSCLC tissues. Therefore, immunohistochemistry for PinX1 was performed following on two independent cohort of NSCLC with complete clinical-pathological and follow-up data. Conventionally, the expression of PinX1 in two cohorts NSCLC patients in keeping with preliminary experiments was frequently suppressed in NSCLC tissues by using a scoring system [20]. The decreased PinX1 staining was found to correlate with smoking condition, Histological Type, T stage, N stage, TNM stage. Moreover, univariate and multivariate analysis in both NSCLC cohort as well as data from the cBioportal for Cancer Genomic demonstrated that PinX1 expression was a strong and independent prognostic indicator for NSCLC disease. Our clinical data analysis indicated that the PinX1 expression might provide useful information in the evaluation prognosis and follow-up schedule guiding for NSCLC patients.

Together, these results propel us to further elucidate a potentially important role of PinX1 as an underlying biological mechanism in the development and/or growth of NSCLC. It has been reported that PinX1 suppress tumor growth and depletion of endogenous PinX1 enhanced tumorigenicity [32]. We observed that knockdown of PinX1 could also enhance cell proliferation and clonogenicity of NSCLC cell lines in vitro. Besides, the capacity of cell inhibition and survival enhancement in PinX1 was also proved in vivo by nude xenograft assay. Several studies discovered that localization of PinX1 extends beyond the tail of telomere, suggesting a novel cell cycle functions outside the telomerase inhibition [14, 15, 33]. Therefore, we analyzed cell cycle in PinX1-slienced and PinX1-overexpression NSCLC cells by EdU cooperation assay and flow cytometry assay. The cell cycle analysis showed that decreased PinX1 expression could accelerate G1 to S phase transition and this acceleration might contribute to NSCLC cells survival ability.

To identify the molecular events involving in PinX1 arrested G1/S transition, we carried out a large-scale proteomic screening using antibody microarray (Full Moon BioSystems) to detect the changes of cell cycle related protein in PinX1-slienced NSCLC cells. Of the 60 cell cycle related proteins, six study-related proteins were differentially observed by 0.2-fold or more (increased: CDK4, CyclinD1, CyclinD2, P-Rb(P-Ser608); decreased: P15^INK4b^, Rb). Subsequently, these screened cell cycle related proteins expressions were confirmed by western blot assay again. The decreased P15 and increased cyclinD1 protein expression lead to retinoblastoma (Rb) phosphorylation, subsequent progression of G1/S phase transition and acceleration of uncontrolled cell proliferation [34]. We suggest that this might be the reason, why there PinX1 correlated with NSCLC tumorigenesis and progression.

According to an mRNA and IncRNA expression profile screening from other group PinX1-related reports, the differentially expressed mRNAs were identified by the fold change in pcDNA3.1-PinX1 group. The fold change of BMP5 mRNA (14.54) was significantly increased in pcDNA3.1-PinX1 group and was expressed at the highest levels. Therefore, we focus on BMP5 to further elucidate the possible molecular mechanism of PinX1’s action via act P15/cyclinD1 pathway [25]. BMP5 was originally identified by their presence in bone-inductive extracts of dematerialized bone and belongs to the transforming growth factor-β (TGF-β) superfamily [35, 36]. It binds and activates TGF-βRI (TGF-β type I receptor) and TGF-βRII (TGF-β type II receptor) and then phosphorylates Smad1/5/8. The phosphorylated Smad1/5/8 together with Smad4 translocated into nucleus and control cycle progression [37]. It has been reported that the mRNA levels of BMP5 was significantly decreased in NSCLC tissues [38]. In this study, the changes of downstream protein BMP5 correlated with PinX1-stimulated cell proliferation. Furthermore, a consistent correlation of PinX1 expression and BMP5 expression in two NSCLC cohorts, as well as the inhibition of noggin and the promotion of ectogenic BMP5 in vitro assay confirmed that BMP5 might be the key molecule contribute to PinX1-inhibited cell proliferation and cell cycle transition.

## Conclusions

We describe, for the first time, the expression pattern of PinX1 in human NSCLC tissues. Our results provide a basis for the concept that decreased expression PinX1 may be important in NSCLC tumorigenesis, and might be a novel predictor for NSCLC patients. Furthermore, the function and mechanistic studies of PinX1 suggest that PinX1-arrested cell cycle transition accounts for the NSCLC’s cell proliferation. In addition, the function of PinX1 in cell cycle transition via BMP5 and P15/cyclinD1 pathway might be responsible for the development and progression of human NSCLC disease.

## Additional files

Additional file 1: Supplementary materials and methods. (DOCX 15 kb)

Additional file 2: Table S1. Area under the receiver-operator curve of PinX1 for each pathological feature in both NSCLC cohorts (DOCX 13 kb)

Additional file 3: Figure S1. Receiver-operator curves (ROC) were used to determine the cut-off score for positive expression of PinX1 protein in both cohorts. The sensitivity and specificity for each outcome were plotted: (A). Gender in learning cohort, (B) M stage in learning cohort, (C) Gender in validation cohort, (D) M stage in validation cohort, (E) T stage in validation cohort, (F) N stage in validation cohort, (G) WHO grade in validation cohort, (H) TNM stage in validation cohort. (TIF 531 kb)

Additional file 4: Figure S2. MTT assay was performed to measure viability of BEAS-2B cells expressing different levels of PinX1. The survival capacity of cells was substantially enhanced in PinX1-silenced BEAS-2B cells (Normal lung epithelial cells). Transfected with PinX1 in BEAS-2B cells displayed a substantial drop in cell viability compared with that of control cells. Each bar represents the mean ± SD of three independent experiments. *, compared to control group (*P*<0.05). (TIF 226 kb)

## Abbreviations

CDK4: Cyclin-dependent kinase 4
CGA: The Cancer Genome Atlas
IHC: Immunohistochemistry
IPP: Image pro plus
NSCLC: Non-small cell lung cancer
qRT-PCR: Quantitative real-time polymerase chain reaction
Rb: Retinoblastoma
ROC: Receiver-operator curve
TRF1: Telomeric repeat binding factor 1
