# Supplementary material for: The depletion of PinX1 involved in the tumorigenesis of non-small cell lung cancer promotes cell proliferation via p15/cyclin D1 pathway
Source: Mol Cancer. 2017 Apr 4;16:74. doi: 10.1186/s12943-017-0637-4 (PMC5379637; doi:10.1186/s12943-017-0637-4)
Supplement: Supplementary file 1 — Supplementary materials and methods. (DOCX 15 kb) [file 12943_2017_637_MOESM1_ESM.docx]

**Supplementary Materials and Methods**

**Immunohistochemistry evaluation**

Semi-quantitative method was used to access the nuclear immunoreactivity of PinX1 protein by evaluating the number of positive tumor cells over the total number of tumor cells. Scores were collected by using 5% increments approach (0%, 5%, 10% … 100%). For the evaluation of BMP5 IHC staining, two sets of indicator containing staining intensity as well as positive areas were recorded by a semi-quantitative scoring criterion. A staining index (values 0–12), obtained as the intensity of BMP5-positive staining (weak, 1; moderate, 2; strong, 3) and the proportion of immune-positive cells of interest (0%, 0; <10%, 1; 10–50%, 2; 51–80%, 3; >80%, 4) were calculated. Finally, the cases were classified into two different groups: low expression cases (score 0–6) and cases with high expression (scores 8–12).

**Selection of cut-off scores.**

As for the cut-off scores for tumor PinX1-positive, receiver–operator curve (ROC) analysis was applied to this decision by using the 0, 1-criterion. ROC was generated by plotting the sensitivity and specificity for each outcome in PinX1 scoring. According to the ROC, the score closest to the point with both maximum sensitivity and specificity (i.e. the point [0.0, 1.0] on the curve) was selected as the cut-off score. For a diagnostic test, the area under ROC curve between 0.7 and 0.9 was deemed as high diagnostic value (Supplementary Table 1). Tumors designated as ‘‘positive’ for the protein were those with scores above the threshold value, whereas negative tumors were considered those with scores below the threshold. However, the clinicopathological features should convert to dichotomization before ROC analysis: Gender (male and female), T stage (early [T1 + T2] or late [T3]), N stage (N0 or N1), M stage (M0 or M1), tumor grade (low [G1 + G2] or high [G3]), and survival (death due to non-small cell lung cancer or censored [lost to follow-up, alive, or death from other causes]). The sensitivity and specificity for each outcome were plotted (Figure 2A and Supplementary Figure 1). PinX1 immunoreactivity was classified by receiver-operator curve (ROC) analysis: (1) low expression defined as less than 65% PinX1 positive cells and (2) high expression defined as greater than 65% PinX1 positive cells.

**Western blotting analysis**

The samples were randomly selected from the learning cohort and treated with ultrasonic processing (5min×3 times) and centrifuged 90min before separated with SDS-PAGE. Then, standard western blotting methods were performed with the following antibodies and dilutions: PinX1 (1:2000, ProteinTech Group, Inc.), GAPDH (1:8000, Santa Cruz Biotechnology), Bcl-2 (1:5000, Cell Signaling Technology), Bax (1:2000, Cell Signaling Technology), P15 (1:1000, Sigma-Aldrich), CDK4 (1:1000, Sigma-Aldrich) , Rb (1:1000, Sigma-Aldrich), p-Rb (1:2000, Sigma-Aldrich)and BMP5 (1:5000, Sigma-Aldrich).
