# Supplementary material for: The depletion of PinX1 involved in the tumorigenesis of non-small cell lung cancer promotes cell proliferation via p15/cyclin D1 pathway
Source: Mol Cancer. 2017 Apr 4;16:74. doi: 10.1186/s12943-017-0637-4 (PMC5379637; doi:10.1186/s12943-017-0637-4)
Supplement: Supplementary file 2 — Area under the receiver-operator curve of PinX1 for each pathological feature in both NSCLC cohorts (DOCX 13 kb) [file 12943_2017_637_MOESM2_ESM.docx]

Supplementary Table 1. Area under the receiver-operator curve of PinX1 for each pathological feature in both NSCLC cohorts.

| Characteristics | AUC(95% Cl) | *P*-value |
| --- | --- | --- |
| Learning cohort |  |  |
| WHO grade | 0.544(0.430-0.657) | 0.450 |
| Gender | 0.570(0.457-0.683) | 0.229 |
| N stage | 0.679(0.573-0.786) | 0.002 |
| TNM stage | 0.744(0.644-0.843) | 0.000 |
| T stage | 0.652(0.544-0.761) | 0.009 |
| M stage | 0.628(0.519-0.737) | 0.028 |
| Validation cohort |  |  |
| WHO grade | 0.550(0.437-0.663) | 0.390 |
| Gender | 0.579(0.466-0.692) | 0.177 |
| N stage | 0.623(0.512-0.733) | 0.035 |
| TNM stage | 0.734(0.635-0.833) | 0.000 |
| T stage | 0.522(0.421-0.633) | 0.045 |
| M stage | 0.639(0.530-0.748) | 0.018 |
| AUC, area under the curve; Cl, confidence interval. | | |
