# Supplementary figures and images for: The depletion of PinX1 involved in the tumorigenesis of non-small cell lung cancer promotes cell proliferation via p15/cyclin D1 pathway
Source: Mol Cancer. 2017 Apr 4;16:74. doi: 10.1186/s12943-017-0637-4 (PMC5379637; doi:10.1186/s12943-017-0637-4)

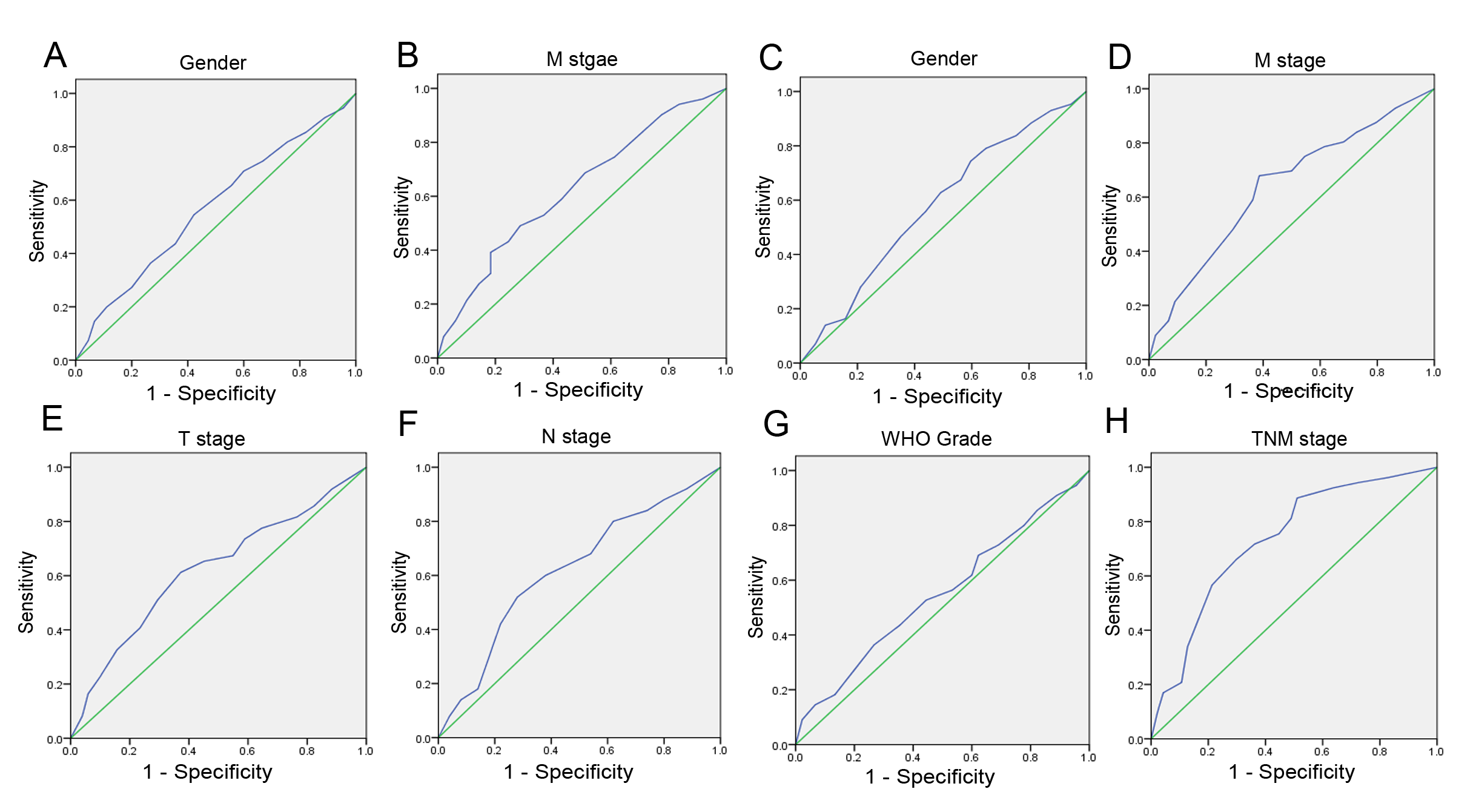

Supplement: Supplementary file 3 — Receiver-operator curves (ROC) were used to determine the cut-off score for positive expression of PinX1 protein in both cohorts. The sensitivity and specificity for each outcome were plotted: (A). Gender in learning cohort, (B) M stage in learning cohort, (C) Gender in validation cohort, (D) M stage in validation cohort, (E) T stage in validation cohort, (F) N stage in validation cohort, (G) WHO grade in validation cohort, (H) TNM stage in validation cohort. (TIF 531 kb) [file 12943_2017_637_MOESM3_ESM.tif]

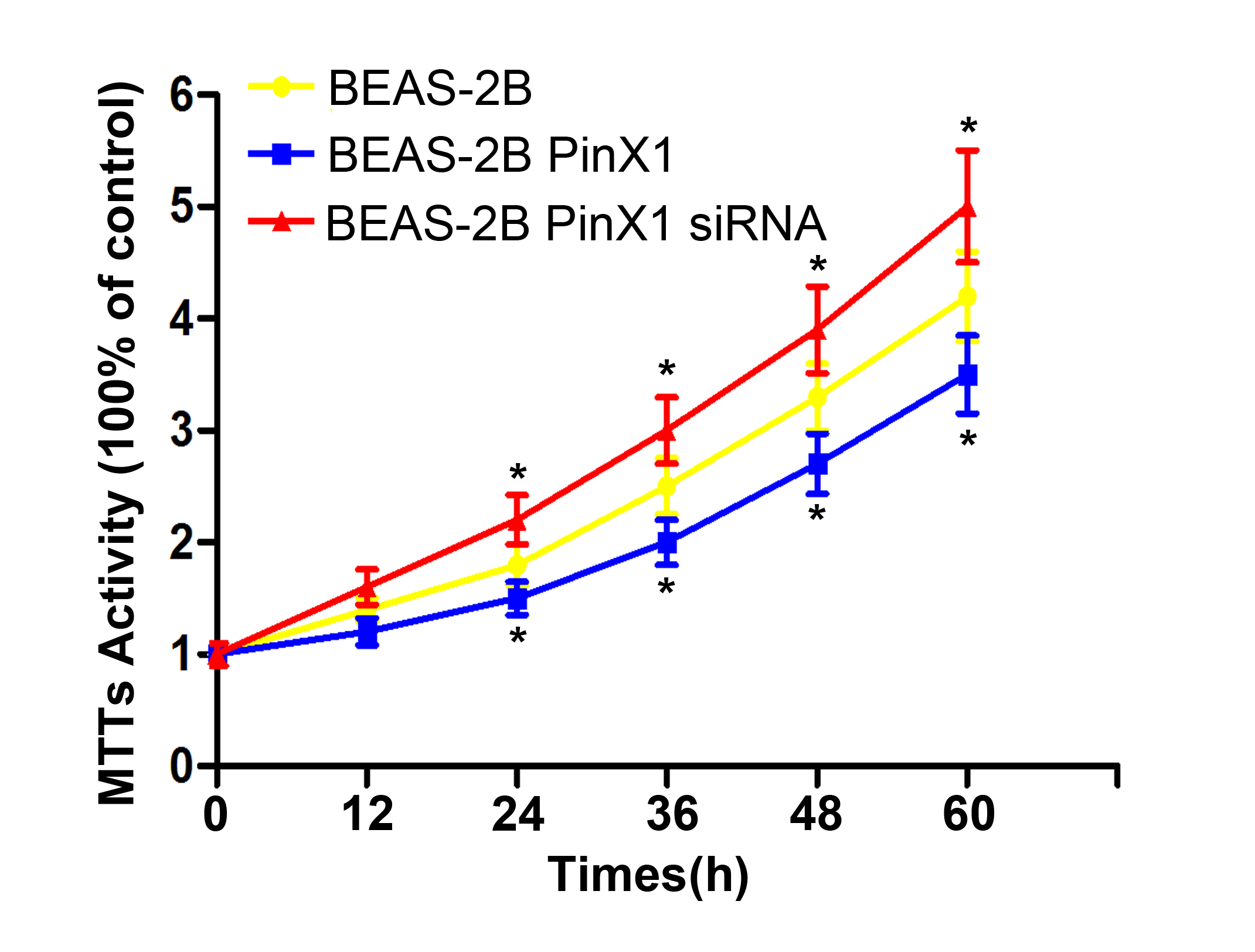

Supplement: Supplementary file 4 — MTT assay was performed to measure viability of BEAS-2B cells expressing different levels of PinX1. The survival capacity of cells was substantially enhanced in PinX1-silenced BEAS-2B cells (Normal lung epithelial cells). Transfected with PinX1 in BEAS-2B cells displayed a substantial drop in cell viability compared with that of control cells. Each bar represents the mean ± SD of three independent experiments. *, compared to control group (P<0.05). (TIF 226 kb) [file 12943_2017_637_MOESM4_ESM.tif]
